# Immunohistochemical selection of biomarkers for tumor-targeted image-guided surgery of myxofibrosarcoma

**DOI:** 10.1038/s41598-020-59735-4

**Published:** 2020-02-19

**Authors:** Jan Marie de Gooyer, Yvonne M. H. Versleijen-Jonkers, Melissa H. S. Hillebrandt-Roeffen, Cathelijne Frielink, Ingrid M. E. Desar, Johannes H. W. de Wilt, Uta Flucke, Mark Rijpkema

**Affiliations:** 1 Department of Radiology and Nuclear Medicine, Radboud university medical center, Nijmegen, the Netherlands; 2 Department of Surgery, Radboud university medical center, Nijmegen, the Netherlands; 3 Department of Medical Oncology, Radboud university medical center, Nijmegen, the Netherlands; 4 Department of Pathology, Radboud university medical center, Nijmegen, the Netherlands

**Keywords:** Diagnostic markers, Sarcoma, Surgical oncology

## Abstract

Myxofibrosarcoma(MFS) is the most common soft tissue sarcoma(STS) in elderly patients. Surgical resection remains the main treatment modality but tumor borders can be difficult to delineate with conventional clinical methods. Incomplete resections are a common problem and local recurrence remains a clinical issue. A technique that has shown great potential in improving surgical treatment of solid tumors is tumor targeted imaging and image-guided surgery with near-infrared fluorescence. To facilitate this technique, it is essential to identify a biomarker that is highly and homogenously expressed on tumor cells, while being absent on healthy non-malignant tissue. The purpose of this study was to identify suitable molecular targets for tumor-targeted imaging of myxofibrosarcoma. Ten potential molecular targets for tumor targeted imaging were investigated with immunohistochemical analysis in myxofibrosarcoma tissue (n = 34). Results were quantified according to the immunoreactive score(IRS). Moderate expression rates were found for uPAR, PDGFRa and EMA/MUC1. High expression rates of VEGF and TEM1 were seen. Strong expression was most common for TEM1 (88.2%). These results confirms that TEM1 is a suitable target for tumor-targeted imaging of myxofibrosarcoma. **Keywords** Image-guided surgery; Immunohistochemistry; Molecular imaging; Myxofibrosarcoma; Soft tissue sarcoma; Tumor endothelial marker 1(TEM1), Vascular endothelial growth factor (VEGF).

## Introduction

Myxofibrosarcoma (MFS) is a histological subtype of soft tissue sarcoma (STS) formerly classified as a myxoid-type malignant fibrous histiocytoma. It has been reclassified and defined as a distinct pathological entity in the World Health Organization (WHO) criteria set in 2002^1,2^. Myxofibrosarcoma is a relatively common sarcoma in the elderly that mainly arises in the extremities^3^. Primary MFS most often presents as a subcutaneous painless, slow growing nodule, but completely infiltrative growth patterns along fascial planes without formation of an evident tumor mass have also been described^4^. The low-grade lesions have a relatively low metastatic potential but show a significant propensity for local recurrences^5–8^. These recurrences show higher-grade histology in up to 50% of all cases compared to the primary tumor^3^. Subsequently, high and intermediate grade disease exhibits a greater metastatic potential, mainly metastasizing to the lungs and lymph nodes^7,9^. Current guidelines recommend wide surgical resection combined with pre or post-operative radiotherapy as the preferred treatment^10^. Despite this strategy, positive margins after surgery occur in up to 20% of cases and reported local relapse rates range from 15 to 65%^5–8,11^.

Currently clinicians rely on conventional imaging modalities such as CT and MRI to determine the location and extent of tumor burden prior to surgery. Translating these conventional imaging modalities to the surgical theatre remains challenging, forcing the surgeon to depend mainly on tactile and visual cues during the procedure. Previous imaging studies have shown that MFS has a tendency to grow in so-called ‘’tail sign” curvilinear extensions that arise from the main tumor^12,13^. These extensions are thought to correlate with microscopic spread of the tumor along fascial planes and have been found extending up to 3 centimeters away from macroscopically visible tumor borders^12,14,15^. Unfortunately, intraoperatieve delineation of these tumor borders and extensions based on tactile and visual clues is arduous and not always possible. Therefore, there is a need for accurate and real-time imaging techniques that can aid in intraoperative detection of disease.

A promising technique that could aid in real-time tumor detection during surgery is tumor-targeted near-infrared (NIR) fluorescence imaging with targeted molecular agents^16^. To facilitate this technique it is essential to identify a biomarker that can be targeted with an imaging agent. This biomarker needs to be abundantly and homogeneously expressed on tumor cells while being absent on benign and healthy tissue. Another prerequisite is that the biomarker’s expression on tumor cells is not significantly altered by preoperative radiotherapy since this is often used for MFS. For a variety of tumor types there are established suitable targets for molecular imaging, but for MFS specific immunohistochemical expression profiles have not yet been described^17–20^.

Therefore, the primary objective of this study was to determine suitable biomarkers for targeted molecular imaging in myxofibrosarcoma using immunohistochemical analyses of surgically obtained tumor specimens. Ten biomarker candidates were selected based on the literature and availability of imaging agents that specifically target these biomarkers^21–29^. The biomarker with the highest expression rate was subsequently evaluated on a selection of whole slides to evaluate the contrast between tumor tissue and adjacent benign regions. The secondary objective was to determine whether the expression of these biomarkers is influenced by preoperative treatment.

## Methods

### Sample selection

Formalin fixed paraffin embedded (FFPE) tissue samples of all patients diagnosed with MFS who underwent surgical resection in the Radboudumc between 2008 and 2015 were reviewed. Patient characteristics are depicted in Supplementary Table 1. The local institutional ethics committee of the Radboud university medical center approved this study. The need for informed consent was waived by the institutional review board since no interventions were performed and the majority of patients were either deceased or lost to follow up (>5 years after initial treatment). All samples and corresponding data were handled and stored anonymously. The study was performed in accordance with the Code of Conduct of the Federation of Medical Scientific Societies in the Netherlands and with the 1964 Helsinki declaration and its later amendments or comparable ethical standards.

### Biomarkers

A total of 10 biomarkers was selected based on literature that reports on tumor targeted imaging of these markers, and the availability of a clinical grade targeting molecule or imaging conjugate (to facilitate future clinical application). The following biomarkers were selected: Human epidermal growth factor receptor 2 (HER-2), carcino-embryonic-antigen (CEA), epithelial cell adhesion molecule (EpCAM), epidermal growth factor receptor (EGFR), epithelial membrane antigen (EMA, also known as MUC-1), urokinase plasminogen activator receptor (uPAR), platelet-derived growth factor receptor α (PDGFRα), vascular endothelial growth factor A (VEGF-A), Tumor endothelial marker 1 (TEM1) and carbonic anhydrase IX (CA-IX).

### Immunohistochemistry on tissue microarrays

A dedicated sarcoma pathologist (UF) at our tertiary referral center reviewed all histological slides and selected regions of interest for the tissue microarrays (TMAs). TMAs with 2 mm cores were sampled and constructed by a dedicated medical technician. Subsequently, TMAs were deparaffinized with xylene, rehydrated in ethanol and rinsed in distilled water according to standard local protocol. Heat-induced antigen retrieval was performed in 10 mM sodium citrate buffer (pH 6.0) or EDTA solution (pH 9.0) for 10 minutes in a microwave or in a PT Module(10 min, 90 C). Endogenous peroxidase activity was blocked with 3% H_2_O_2_ in 10Mm phosphate buffered saline (PBS) for 10 min at room temperature. Subsequently, tissue sections were washed with 10 mM PBS and stained with primary antibodies at room temperature or 4 °C respectively. Primary antibodies used and their characteristics are depicted in Supplementary Table 2. Next, tissue sections were incubated with Poly-HRP-GAMs/Rb IgG (ImmunoLogic) in EnVision™ FLEX Wash Buffer (Dako A/S, Denmark) (1:1) for 30 min at room temperature. Antibody binding was visualized using the EnVision™ FLEX Substrate Working Solution (Dako) for 10 min at room temperature. The slides stained with VEGF, EGFR and CAIX were visualized with DAB bright (immunologic) for 8 minutes at room temperature. All sections were counterstained with haematoxylin for 5 seconds, dehydrated in ethanol and coverslipped. uPAR sections were treated similarly except that epitope retrieval was performed using PT link and a low-pH Envision FLEX target retrieval solution (Agilent, Santa Clara, United States) and visualization was done with Envision anti mouse (K4001, Agilent) and 3,3 diaminobenzidine tetrahydrochloride (Agilent). For HER-2, a ready to use HercepTest kit was used (Dako) according to the manufacturer’s protocol. Appropriate positive controls were stained for all primary antibodies.

### Immunohistochemical scoring method

**Figure 1:**
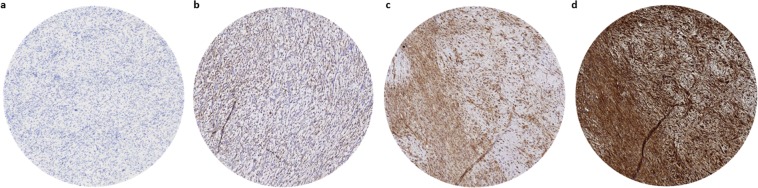
Examples of expression scores. (**a**) No expression (CEA), (**b**) low expression (PDGFRa), (**c**) moderate expression (VEGF), (**d**) strong expression (TEM1).

The extent of biomarker expression was assessed in primary tumor tissue on a scale from 0–4. (0 =  <10% positive cells, 1 = 10–25% positive cells, 2 = 25–50% positive cells, 3 = 50–75% positive cells, 4 =  >75% positive cells. Biomarker expression intensity was scored on a scale ranging from 0 to 3 (0 for no staining, 1 for weak staining, 2 for moderate staining and 3 for strong staining). For this study we combined the percentage and intensity scores into a final expression score according to the immunoreactive score(IRS) scoring method^30^. The IRS is calculated as follows: Final expression score = percentage of positive cells multiplied by the staining intensity. This results in a final score ranging from 0–12. The final expression score was divided into 4 categories (0–1 = no expression, 2–3 = weak expression, 4–8 = moderate expression, 9–12 = strong expression). Staining was scored on membranous expression, except for VEGF which was scored on cytoplasmic expression. Strong biomarker expression was considered as highly suitable for tumor-targeted imaging and image-guided surgery purposes. Evaluation of biomarker expression was performed independently by 2 trained observers, one dedicated sarcoma pathologist and one assistant professor of sarcoma research (U.F. and Y.V.). All cases where inter-observer disagreement occurred were discussed together with a third observer (J.M.G) until agreement was reached on the final expression score. Representative images of these expression intensity scores are depicted in Fig. 1.

### Negative controls and tumor borders

All FFPE blocks selected for the TMAs were evaluated to select those that represented a section of MFS with a clearly distinguishable border between malignant and benign/preexistent tissue. 4 µm sections were stained for H&E and the biomarker with the highest rate of expression found in the previous experiment. Tumor borders and invasive extensions were defined and marked by a dedicated sarcoma pathologist (U.F.) on the H&E slide. The immunostaining on tumor versus various types of benign tissue was assessed by the pathologist.

### Statistical analysis

Descriptive statistics were calculated and shown for expression of all biomarkers. Samples were also divided in 2 groups, those that were treated with or without neoadjuvant radiotherapy. Differences in biomarker expression between these groups were calculated and compared using the Mann-Whitney U test. Results were considered statistically significant when the p value was smaller than 0.05. All statistical analyses were performed using the SPSS (version 23; IBM, Chicago, IL, USA) and GraphPad Prism 6 (GraphPad Software Inc., La Jolla, CA, USA).

### Ethics approval

This study was conducted according to the ethical standards laid down in the 1964 Declaration of Helsinki and in accordance with the ethical standards of the local ethics committee and with national Dutch guidelines (“Code for Proper Secondary Use of Human Tissues,” Dutch Federation of Medical Scientific Societies). The local medical ethics committee at the Radboud university medical center, Nijmegen, the Netherlands, approved the study prior to its conduction. The need for informed consent was waived by the institutional review board.

## Results

### Biomarker expression

**Figure 2:**
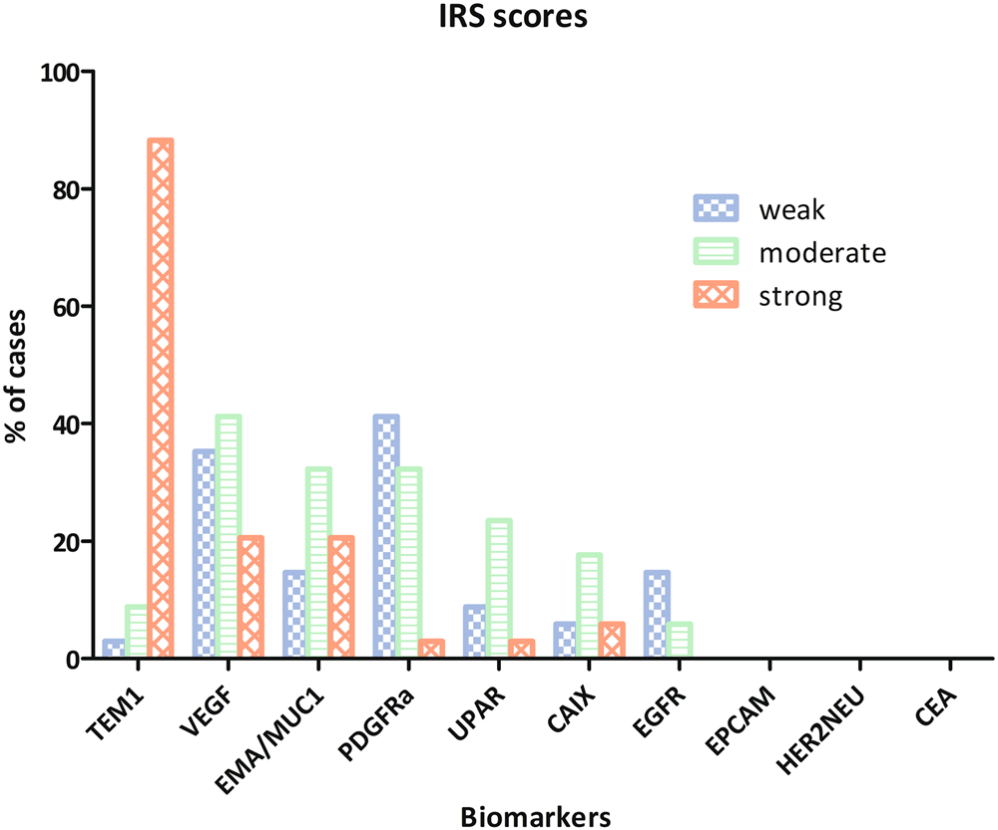
IRS Expression scores.

Cases with inconclusive pathology results were re-evaluated. In five cases the diagnosis MFS was not certain and these were excluded from further analysis. A total of 34 cases with proven diagnosis of MFS were included in the TMA and staining protocol. Ten of these 34 cases were treated with preoperative radiotherapy Per case a minimum of 2 and a maximum of 3 samples per FFPE block were taken and processed in the TMAs. All cases expressed TEM1, with strong expression being present in 30 cases (88.2%). For VEGF, 33 (93%) of all cases showed expression but only 7 (20.6%) exhibited strong expression. EMA/MUC-1 staining was seen in 22 (57.6%) cases with 7 (20.6%) exhibiting strong expression. Only 2(5.9%) of the 10 cases that stained positive for CAIX demonstrated strong expression. Expression of PDGFRa was observed in 26(76.5%) cases but strong expression occurred only once. 12(25.3%) cases stained positive for UPAR but strong expression was seen in 1(2.9%) case. EGFR expression was observed in 9 cases (26.5%) but no strong expression was seen. None of the cases demonstrated expression of EpCAM, CEA or HER2NEU. The expression rates and corresponding percentages of all tumor markers are depicted in Fig. 2.

### Preoperative treatment effects

Higher rates of strong expression after preoperative treatment with radiotherapy were seen for TEM1 but this was not statistically significant (p = 0.176). The expression pattern of EMA/MUC1 seems to be negatively influenced by preoperative treatment but this was also not statistically significant (p = 0.181). No significant effect of neoadjuvant radiotherapy on biomarker expression was observed in this cohort. Results of treatment effect are shown in Table 1.

**Table 1:** Treatment effect on scoring intensity.

	Neoadjuvant radiotherapy (n = 10)		Moderate	Strong	Surgery without radiotherapy (n = 24)		Moderate	Strong	p-value
	None	Weak			None	Weak			
CEA n (%)	10 (100)	0 (0)	0 (0)	0 (0)	24 (100)	0 (0)	0 (0)	0 (0)	1
EPCAM n (%)	10 (100)	0 (0)	0 (0)	0 (0)	24 (100)	0 (0)	0(0)	0 (0)	1
HER2NEU n (%)	10 (100)	0 (0)	0 (0)	0 (0)	24 (100)	0 (0)	0 (0)	0 (0)	1
EGFR n (%)	7 (70)	1 (10)	2 (20)	0 (0)	18 (75)	4 (16.7)	2 (8.3)	0 (0)	0.660
CAIX n (%)	8 (80)	1 (10)	0 (0)	1 (10)	16 (66.7)	1 (4.2)	6 (25.0)	1 (4.2)	0.451
UPAR n (%)	7 (70)	1 (10)	2 (20)	0 (0)	15 (62.5)	2 (8.3)	6 (25.0)	1 (4.2)	0.607
PDGFRa n (%)	2 (20)	5 (50)	3 (30)	0 (0)	6 (25.0)	9 (37.5)	8 (33.3)	1 (4.2)	0.856
EMAMUC1 n(%)	4 (40)	3 (30)	2 (20)	1 (10)	7 (29.2)	2 (8.3)	9 (37.5)	6 (25.0)	0.181
VEGF n (%)	0 (0)	4 (40)	5 (50)	1 (10)	1 (4.2)	8 (33.3)	9 (37.5)	6 (25.0)	0.643
TEM1 n (%)	0 (0)	0 (0)	0 (0)	10 (100)	0 (0)	1 (4.2)	3 (12.5)	20 (83.3)	0.176

IRS scores, categorized by type of treatment. P-values: Mann whitney U test.

### Negative controls and tumor borders

**Figure 3:**
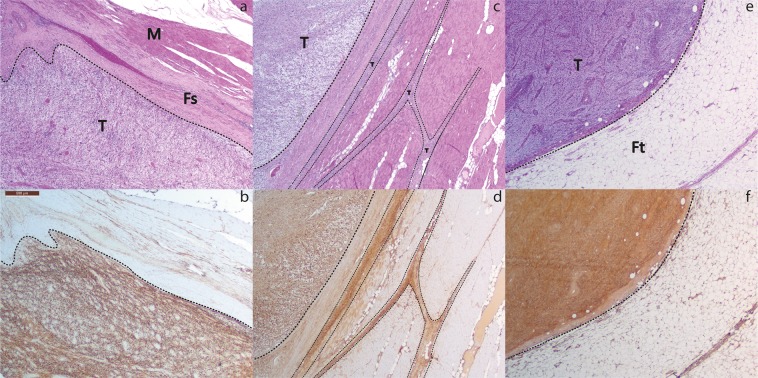
(**a**) H&E staining of MFS and adjacent fascia and muscle. (**b**) Corresponding TEM1 staining shows strong TEM1 expression in MFs and virtually no TEM1 expression in adjacent fascia and muscle. (**c**) H&E staining of a MFS bordering and muscle tissue. Infiltrative growth along fatty septa is clearly visible. (**d**) Corresponding TEM1 staining shows clear contrast between TEM1 expressing MFS and bordering muscular tissue. Microscopic tumor extensions in the septa show strong TEM1 expression. (**e**) H&E staining of a MFS growing into subcutaneous fat. (**e**) Corresponding TEM1 staining shows strong expression of TEM1 in MFS and clear contrast with TEM1 negative adjacent subcutaneous fat. ***Dashed lines indicate tumor borders, T = Tumor, Fs = Fascia, M = Muscle, Ft = Fat.

Ten FFPE blocks showed clear tumor to benign tissue borders and were selected for further analysis. Tissue sections of 4 µm were stained for H&E and TEM1. All sections showed invasive MFS next to various benign/preexistent tissues such as subcutaneous fat, skeletal muscle and adjacent fascia, epidermis, peripheral nerve bundles, blood vessels and necrotic tumor regions. Clear contrast was seen between MFS and benign/preexistent tissue in all sections stained with TEM1. Muscle, fascia and subcutaneous fat showed no discernable TEM1 expression. Weak expression was seen in normal endothelium and fibrous tissue but strong contrast to tumor tissue was still present. Two sections revealed microscopic tumor involvement of the septa highlighted by strong TEM1 expression. Three representative examples are shown in Fig. 3.

## Discussion

In this immunohistochemical study the expression of 10 biomarker candidates in MFS was examined in surgical samples of 34 patients. Moderate expression rates were observed for uPAR, PDGFRa and EMA/MUC1 and high expression rates of VEGF and TEM1 were observed. Strong overexpression of TEM1 was observed in 89% of all cases, demonstrating great potential as a biomarker for targeted approaches for diagnosis and treatment.

Targeted NIR fluorescence imaging has already been studied in several clinical trials for colorectal, head and neck, pancreatic and renal cancer and has shown its potential and added value in intraoperative decision making^21,25,31–33^. As a result of these trials, a variety of suitable biomarkers have been identified for NIR-fluorescence guided surgery. For soft tissue sarcoma, targeted NIR- fluorescence-guided surgery is still mostly uncharted territory and the literature on tumor marker expression is very limited, especially for MFS.

Small studies performing expanded molecular profiling of MFS are described in the literature but immunohistochemical studies that specifically investigate potential targets for tumor-targeted imaging are lacking^34^. Scoccianti *et al*.^35^ reported on EMA/MUC1 expression in MFS and found no positive staining for EMA/MUC-1. The difference of the results reported in this manuscript might be attributed to a variety of factors such as the use of different antigen retrieval techniques and the use of a different primary antibody. Forker *et al*.^36^ investigated a selection of hypoxia markers, including CAIX, in a large cohort of soft tissue sarcomas. The cohort also contained 52 myxofibrosarcomas but the CA-IX expression was only reported for all STS types together. Mentzel *et al*.^37^ studied vascularity and tumor progression in a series containing 43 MFS cases. They used mRNA *in situ* hybridization (ISH) to study VEGF expression and report that all cases showed some extent of VEGF expression. This is in accordance with our results, but regrettably, VEGF expression was only studied in 7 of their 43 cases. Sato *et al*.^38^ studied the expression of EGFR and ERB2(HER2NEU) in Japanese myxofibrosarcoma cases. They did not find HER2NEU expression but report that 98% of cases shows EGFR expression. This is a surprising lack of compatibility with the EGFR expression rates in our cohort, where EGFR expression was only found in 26.5%. Because of this discrepancy, an additional EGFR staining with a different primary antibody was performed in our cohort but again no EGFR expression was found. The difference might be explained by genetic differences, the use of a different primary antibody or variability in antigen retrieval techniques used.

The suitability of a biomarker for MFS is not solely dependent on its upregulation but influenced by several factors. A scoring system to objectify the assessment of biomarker suitability for targeted NIR-Fluorescence image-guided surgery has been proposed by Oosten *et al*.^39^. This TASC scoring system corrects for a variety of factors, such as extracellular localization of the biomarker, internalization of the target, previous use of the biomarker in *in vivo* imaging studies and enzymatic activity in or around tumor tissue. The final maximum TASC score is 22 points and a score ≥18 is considered suitable for imaging purposes. When applying the TASC criteria to TEM1, the final score of 21 confirms its high suitability as a target for NIR fluorescence-guided surgery. The second best marker in our cohort (VEGF) is an interesting and applicable target for NIR-Fluorescence image-guided surgery since a fluorescently-labeled tracer targeting VEGF (bevacuzimab-IRDye800CW) is clinically available and has been extensively studied for several malignancies^32,40^. The main drawback of VEGF is that it is a signal protein produced to stimulate the formation of blood vessels. Therefore the protein may also be found in benign tissue, especially in injured regions with high rates of angiogenesis^41^. Because of some of these characteristics, the final TASC score of VEGF is 17, which is just below the threshold of 18 set by Oosten *et al*.^39^.

The absence of the biomarker in benign tissue mentioned in these TASC criteria remains one of the most pivotal characteristics of a suitable biomarker. TEM1 immunohistochemistry on complete tissue sections shows that expression was absent or very limited on a variety of benign/preexistent tissues such as subcutaneous fat, epidermis, muscle and fascia while the primary tumors with diffuse infiltration along preexisting fibrous septa was strongly positive for TEM1. This is of great clinical importance since MFS very frequently extends along the septa into resection margins, with one third of all MFS tumors being larger than originally expected during surgical resection^42^. The fact that muscular fascia did not show TEM1 expression is also of interest because it suggests that TEM1 targeted fluorescence imaging can allow the surgeon to assess if there is tumor involvement of the fascia. The surgeon can follow the muscular fascia as a surgical plane to guide the resection when tumor involvement is not detected. Peripheral nerve bundles were also negative for TEM1, allowing for identification and possible sparing of vital nerves during procedures and therefore reduce surgical morbidity.

A potential advantage of a fluorescent agent targeting TEM1 is that it may be used for a variety of oncological indications. The development of clinical grade tracers for tumor-targeted NIR-fluorescence imaging is an expensive and lengthy process so ideally the developed agent targets a biomarker that is (over)expressed on multiple tumor types. TEM1 is a target with such characteristics since it is strongly expressed on tumor cells, tumor vasculature and stroma in the majority of soft tissue and bone sarcomas^43,44^. While TEM1 is known to be expressed on tumor cells of several sarcoma subtypes^44^, the expression patterns in other cancer types are generally of a different nature. In melanoma, ovarian, breast, lung and brain cancers TEM1 is mainly found to be highly expressed on perivascular and stroma cells^44–48^. This does not mean that targeting TEM1 is not feasible for these cancer types because stromal targeting also yields potential for image-guided surgery^19^. Besides intraoperative imaging, TEM1 is also an attractive target for preoperative imaging. A preclinical trial investigating TEM1-targeted ^89^Zr-Immuno-PET with the clinical grade monoclonal antibody ontuxizumab has shown the possibility to determine TEM1 status in a murine model^49^. This could potentially facilitate non-invasive selection of patients suitable for TEM1 targeted NIR- Fluorescence image-guided surgery, systemic therapy with antibodies or antibody-drug-conjugates^26,44^. TEM1 targeted immuno-PET may also have potential as a diagnostic tool for metastases detection of STS, since these retain TEM1 expression^50^.

The present study contains several limitations. First, the analyzed cohort is relatively small because MFS is a rare disease. Therefore the analysis of preoperative treatment effect on expression patterns lacks sufficient statistical power. Nevertheless, to our knowledge this study is the first cohort of patients with MFS analyzed for biomarkers for tumor-targeted imaging. Second, the selection of biomarkers was not completely comprehensive, also because selection was partially based on availability of clinical grade targeting agents or imaging conjugates. Finally, because TMAs were used for staining purposes, adjacent benign tissue such as muscle, fat or fascia was not investigated for all markers. However, the marker with the highest expression (TEM1) was extensively investigated for expression in a variety of benign adjacent tissues, emphasizing its potential as a biomarker for image-guided surgery. These results are supported by the literature reporting on the absence of TEM1 expression in benign and healthy tissue^50,51^.

Tumor -targeted image-guided surgery has the potential to improve surgical treatment of myxofibrosarcoma. In this immunohistochemical study we investigated a variety of biomarker candidates to determine a suitable target for NIR fluorescence image-guided surgery of myxofibrosarcoma. The results clearly show that TEM1 is an excellent potential biomarker to investigate in future studies.

## Supplementary information

Supplementary Dataset 1.

## References

[1] Angervall L, Kindblom LG, Merck C (1977). Myxofibrosarcoma. A study of 30 cases. *Acta pathologica et microbiologica Scandinavica*. Section A, Pathology.

[2] Fletcher CD (2006). The evolving classification of soft tissue tumours: an update based on the new WHO classification. Histopathology.

[3] Willems SM, Debiec-Rychter M, Szuhai K, Hogendoorn PC, Sciot R (2006). Local recurrence of myxofibrosarcoma is associated with increase in tumour grade and cytogenetic aberrations, suggesting a multistep tumour progression model. Modern pathology: an official journal of the United States and Canadian Academy of Pathology, Inc.

[4] Mentzel T (1996). Myxofibrosarcoma. Clinicopathologic analysis of 75 cases with emphasis on the low-grade variant. The American journal of surgical pathology.

[5] Look Hong NJ (2013). Prognostic factors and outcomes of patients with myxofibrosarcoma. Annals of surgical oncology.

[6] Huang HY, Lal P, Qin J, Brennan MF, Antonescu CR (2004). Low-grade myxofibrosarcoma: a clinicopathologic analysis of 49 cases treated at a single institution with simultaneous assessment of the efficacy of 3-tier and 4-tier grading systems. Hum Pathol.

[7] Haglund KE (2012). Recurrence patterns and survival for patients with intermediate- and high-grade myxofibrosarcoma. International journal of radiation oncology, biology, physics.

[8] Sanfilippo R (2011). Myxofibrosarcoma: prognostic factors and survival in a series of patients treated at a single institution. Annals of surgical oncology.

[9] Tsuchie H (2017). Distant metastasis in patients with myxofibrosarcoma. Upsala journal of medical sciences.

[10] Casali PG (2018). Soft tissue and visceral sarcomas: ESMO-EURACAN Clinical Practice Guidelines for diagnosis, treatment and follow-up. Annals of oncology: official journal of the European Society for Medical Oncology/ESMO.

[11] Odei B (2018). Predictors of Local Recurrence in Patients With Myxofibrosarcoma. American journal of clinical oncology.

[12] Lefkowitz RA (2013). Myxofibrosarcoma: prevalence and diagnostic value of the “tail sign” on magnetic resonance imaging. Skeletal radiology.

[13] Waters B (2007). Low-grade myxofibrosarcoma: CT and MRI patterns in recurrent disease. AJR. American journal of roentgenology.

[14] Ghazala CG (2016). Myxofibrosarcoma of the extremity and trunk: a multidisciplinary approach leads to good local rates of LOCAL control. The bone & joint journal.

[15] Yoo HJ (2014). MR imaging of myxofibrosarcoma and undifferentiated sarcoma with emphasis on tail sign; diagnostic and prognostic value. European radiology.

[16] Vahrmeijer AL, Hutteman M, van der Vorst JR, van de Velde CJ, Frangioni JV (2013). Image-guided cancer surgery using near-infrared fluorescence. Nature reviews. Clinical oncology.

[17] Hoogstins CE (2017). In Search for Optimal Targets for Intraoperative Fluorescence Imaging of Peritoneal Metastasis From Colorectal Cancer. Biomarkers in cancer.

[18] Boogerd LS (2018). Biomarker expression in rectal cancer tissue before and after neoadjuvant therapy. Onco Targets Ther.

[19] Boonstra MC (2015). Stromal Targets for Fluorescent-Guided Oncologic. Surgery. Frontiers in oncology.

[20] Boonstra MC (2016). Selecting Targets for Tumor Imaging: An Overview of Cancer-Associated Membrane Proteins. Biomarkers in cancer.

[21] Boogerd, L. S. F. *et al*. Safety and effectiveness of SGM-101, a fluorescent antibody targeting carcinoembryonic antigen, for intraoperative detection of colorectal cancer: a dose-escalation pilot study. *The lancet. Gastroenterology & hepatology* **3**, 181–191, doi:10.1016/s2468-1253(17)30395-3 (2018). 10.1016/S2468-1253(17)30395-3 29361435

[22] Terwisscha van Scheltinga AG (2011). Intraoperative near-infrared fluorescence tumor imaging with vascular endothelial growth factor and human epidermal growth factor receptor 2 targeting antibodies. Journal of nuclear medicine: official publication, Society of Nuclear Medicine.

[23] Boogerd LSF (2019). Fluorescence-guided tumor detection with a novel anti-EpCAM targeted antibody fragment: Preclinical validation. Surg Oncol.

[24] Chen H (2015). MUC1 aptamer-based near-infrared fluorescence probes for tumor imaging. Molecular imaging and biology: MIB: the official publication of the Academy of Molecular Imaging.

[25] Hekman MC (2018). Tumor-targeted Dual-modality Imaging to Improve Intraoperative Visualization of Clear Cell Renal Cell Carcinoma: A First in Man Study. Theranostics.

[26] Li C (2014). Development, optimization, and validation of novel anti-TEM1/CD248 affinity agent for optical imaging in cancer. Oncotarget.

[27] Nishio, N. *et al*. Optimal Dosing Strategy for Fluorescence-Guided Surgery with Panitumumab-IRDye800CW in Head and Neck Cancer. *Molecular imaging and biology: MIB: the official publication of the Academy of Molecular Imaging*, 10.1007/s11307-019-01358-x (2019). 10.1007/s11307-019-01358-x PMC7017887 31054001

[28] Skovgaard D (2017). Safety, Dosimetry, and Tumor Detection Ability of (68)Ga-NOTA-AE105: First-in-Human Study of a Novel Radioligand for uPAR PET Imaging. Journal of nuclear medicine: official publication, Society of Nuclear Medicine.

[29] Dissoki S, Abourbeh G, Salnikov O, Mishani E, Jacobson O (2015). PET molecular imaging of angiogenesis with a multiple tyrosine kinase receptor-targeted agent in a rat model of myocardial infarction. Molecular imaging and biology: MIB: the official publication of the Academy of Molecular Imaging.

[30] Specht E (2015). Comparison of immunoreactive score, HER2/neu score and H score for the immunohistochemical evaluation of somatostatin receptors in bronchopulmonary neuroendocrine neoplasms. Histopathology.

[31] Hoogstins CES (2018). Image-Guided Surgery in Patients with Pancreatic Cancer: First Results of a Clinical Trial Using SGM-101, a Novel Carcinoembryonic Antigen-Targeting, Near-Infrared Fluorescent Agent. Annals of surgical oncology.

[32] Harlaar NJ (2016). Molecular fluorescence-guided surgery of peritoneal carcinomatosis of colorectal origin: a single-centre feasibility study. *The lancet*. Gastroenterology & hepatology.

[33] Miller SE (2018). First-in-human intraoperative near-infrared fluorescence imaging of glioblastoma using cetuximab-IRDye800. Journal of neuro-oncology.

[34] Heitzer E (2017). Expanded molecular profiling of myxofibrosarcoma reveals potentially actionable targets. Modern pathology: an official journal of the United States and Canadian Academy of Pathology, Inc.

[35] Scoccianti G (2016). Soft tissue myxofibrosarcoma: A clinico-pathological analysis of a series of 75 patients with emphasis on the epithelioid variant. Journal of surgical oncology.

[36] Forker L (2018). The hypoxia marker CAIX is prognostic in the UK phase III VorteX-Biobank cohort: an important resource for translational research in soft tissue sarcoma. Br J Cancer.

[37] Mentzel T (2001). The association between tumour progression and vascularity in myxofibrosarcoma and myxoid/round cell liposarcoma. Virchows Archiv: an international journal of pathology.

[38] Sato O (2005). Expression of epidermal growth factor receptor, ERBB2 and KIT in adult soft tissue sarcomas: a clinicopathologic study of 281 cases. Cancer.

[39] van Oosten M, Crane LM, Bart J, van Leeuwen FW, van Dam GM (2011). Selecting Potential Targetable Biomarkers for Imaging Purposes in Colorectal Cancer Using TArget Selection Criteria (TASC): A Novel Target Identification Tool. Translational oncology.

[40] Tjalma JJ (2016). Molecular Fluorescence Endoscopy Targeting Vascular Endothelial Growth Factor A for Improved Colorectal Polyp Detection. Journal of nuclear medicine: official publication, Society of Nuclear Medicine.

[41] Ferrara N, Davis-Smyth T (1997). The biology of vascular endothelial growth factor. Endocrine reviews.

[42] Folpe, A. L. “Hey!- Whatever Happened to Hemangiopericytoma and Fibrosarcoma?” An Update on Selected Conceptual Advances in Soft Tissue Pathology Which Have Occurred Over the Past 50 Years. *Hum Pathol*, 10.1016/j.humpath.2019.10.001 (2019). 10.1016/j.humpath.2019.10.001 31669060

[43] Rouleau C (2008). Endosialin protein expression and therapeutic target potential in human solid tumors: sarcoma versus carcinoma. Clinical cancer research: an official journal of the American Association for Cancer Research.

[44] Guo Y (2018). Tumour endothelial marker 1/endosialin-mediated targeting of human sarcoma. European journal of cancer (Oxford, England: 1990).

[45] Christian S (2008). Endosialin (Tem1) is a marker of tumor-associated myofibroblasts and tumor vessel-associated mural cells. The American journal of pathology.

[46] Kiyohara E (2015). Endosialin Expression in Metastatic Melanoma Tumor Microenvironment Vasculature: Potential Therapeutic Implications. Cancer microenvironment: official journal of the International Cancer Microenvironment Society.

[47] Simonavicius N (2008). Endosialin (CD248) is a marker of tumor-associated pericytes in high-grade glioma. Modern pathology: an official journal of the United States and Canadian Academy of Pathology, Inc.

[48] Davies G, Cunnick GH, Mansel RE, Mason MD, Jiang WG (2004). Levels of expression of endothelial markers specific to tumour-associated endothelial cells and their correlation with prognosis in patients with breast cancer. Clinical & experimental metastasis.

[49] Lange SE (2016). Development of 89Zr-Ontuxizumab for *in vivo* TEM-1/endosialin PET applications. Oncotarget.

[50] O’Shannessy DJ (2016). Endosialin and Associated Protein Expression in Soft Tissue Sarcomas: A Potential Target for Anti-Endosialin Therapeutic Strategies. Sarcoma.

[51] Teicher BA (2019). CD248: A therapeutic target in cancer and fibrotic diseases. Oncotarget.

