# Supplementary material for: Immunohistochemical selection of biomarkers for tumor-targeted image-guided surgery of myxofibrosarcoma
Source: Sci Rep. 2020 Feb 19;10:2915. doi: 10.1038/s41598-020-59735-4 (PMC7031512; doi:10.1038/s41598-020-59735-4)
Supplement: Supplementary file 1 — Supplementary Dataset 1. [file 41598_2020_59735_MOESM1_ESM.docx]

**Immunohistochemical selection of biomarkers for tumor-targeted image-guided surgery of myxofibrosarcoma**Jan Marie de Gooyer^1,2^, Yvonne M. H. Versleijen-Jonkers^3^, Melissa H. S. Hillebrandt-Roeffen^3^, Cathelijne Frielink^1^, Ingrid M. E. Desar^3^, Johannes H. W. de Wilt^2^, Uta Flucke^4^, Mark Rijpkema^1^
*^1^ Department of Radiology and Nuclear Medicine, Radboud university medical center, Nijmegen the Netherlands, ^2^ Department of Surgery, Radboud university medical center, Nijmegen the Netherlands, ^3^ Department of Medical Oncology, Radboud university medical center, Nijmegen the Netherlands, ^4^ Department of Pathology, Radboud university medical center, Nijmegen the Netherlands*

**Running head:** Biomarker selection for image-guided surgery of myxofibrosarcoma.
Original article
 **Corresponding author contact information:***Jan-Marie de Gooyer, MD
Department of Radiology and Nuclear Medicine (618)
PO Box 9101
6500 HB Nijmegen, the Netherlands
Phone: +31 (0)24 366 72 44
Email: jan-marie.gooyer@radboudumc.nl
Fax: 0031 24 363 51 15*

**Supplementary table 1 Case characteristics**

| Case | Gender | Age | Tumor location | Tumor grade | Preoperative therapy |
| --- | --- | --- | --- | --- | --- |
| 1 | Female | 81 | Trunk | 3 |  |
| 2 | Female | 76 | Lower limb | 3 |  |
| 3 | Male | 57 | Trunk | 2 | Radiotherapy |
| 4 | Male | 44 | Lower limb | 3 |  |
| 5 | Male | 67 | Lower limb | 3 |  |
| 6 | Female | 67 | Upper limb | unknown |  |
| 7 | Male | 70 | Lower limb | 3 | Radiotherapy |
| 8 | Female | 69 | Lower limb | 3 | Radiotherapy |
| 9 | Male | 69 | Lower limb | 3 | Radiotherapy |
| 10 | Female | 67 | Trunk | 3 | Radiotherapy |
| 11 | Male | 78 | Lower limb | 3 | Radiotherapy |
| 12 | male | 52 | Lower limb | 2/3 |  |
| 13 | Male | 68 | Lower limb | 3 | Radiotherapy |
| 14 | Female | 48 | Lower limb | 3 |  |
| 15 | Male | 67 | Lower limb | unknown | Radiotherapy |
| 16 | Male | 75 | Upper limb | 3 | Radiotherapy |
| 17 | Male | 70 | Lower limb | 3 |  |
| 18 | Female | 75 | Lower limb | 2 |  |
| 19 | Male | 62 | Lower limb | 2 |  |
| 20 | Male | 67 | Lower limb | 3 | Radiotherapy |
| 21 | Male | 65 | Lower limb | 3 |  |
| 22 | Female | 61 | Lower limb | 3 |  |
| 23 | Male | 65 | Upper limb | 2 |  |
| 24 | Male | 66 | Lower limb | 3 |  |
| 25 | Female | 62 | Upper limb | 3 |  |
| 26 | Female | 65 | Lower limb | 3 |  |
| 27 | Female | 74 | Upper limb | 2 |  |
| 28 | Male | 67 | Lower limb | 3 |  |
| 29 | Male | 61 | Lower limb | 3 |  |
| 30 | Male | 67 | Upper limb | 1 |  |
| 31 | Female | 45 | Lower limb | 3 |  |
| 32 | Female | 56 | Lower limb | 3 |  |
| 33 | Female | 75 | Lower limb | 3 |  |
| 34 | Male | 79 | Lower limb | 3 |  |

**Supplementary table 2, primary antibodies**

| **Antigen** | **Source** | **Clone** | **Manufacturer** | **Cat nr** | **Dilution** | **Retrieval buffer** | **Incubation time** | **Incubation temperature** |
| --- | --- | --- | --- | --- | --- | --- | --- | --- |
| CEA | Mouse monoclonal | COL-1 | Immunologic | ILM 83111-CO1 | 1:400 | EDTA | 1 hr | RT |
| HER2NEU | Ready-to-use HercepTest™ kit | - | DAKO | K5204 | - | - | - | - |
| EpCAM | Mouse monoclonal | VU-1D9 | Immunologic | VWRKILM 6727-C1 | 1:200 | Citrate | 1 hr | RT |
| EGFR | Rabbit monoclonal | D38B1XP | Cell signaling technology | 4267 | 1:300 | Citrate | 1 hr | RT |
| EMA (MUC1) | Mouse monoclonal | E29 | DAKO | M061301 | 1:250 | EDTA | 1 hr | RT |
| uPAR | Mouse monoclonal | ATN-617 | - | - | 1:400 | Low pH PT Module (Agilent) | o/n | RT |
| VEGF-A | Mouse monoclonal | G153-694 | BD Pharmingen | 555036 | 1:100 | No antigen retrieval done | 1hr | RT |
| CA-IX | Mouse monoclonal | -- |  | - | 1/10000 | Citrate | 1hr | RT |
| PDGFRa | Rabbit monoclonal | D13C6 | Cell signalling technology | #5241 | 1:200 | EDTA | o/n | 4°C |
| TEM1 | Rabbit  monoclonal | EPR17081 | Abcam | Ab204914 | 1:2000 | EDTA | o/n | 4°C |
